# Circulating tumor cells (CTCs) and *hTERT* gene expression in CTCs for radiotherapy effect with lung cancer

**DOI:** 10.1186/s12885-023-10979-z

**Published:** 2023-05-24

**Authors:** Ying Xu, Xue Ren, Tong Jiang, Shuang Lv, Kuanke Gao, Yunen Liu, Ying Yan

**Affiliations:** 1 Department of Radiation Oncology, General Hospital of Northern Theater Command, No. 83 Wenhua Road, Shenhe District, Shenyang, l10016 China; 2 Shandong Province Heze Municipal Hospital, Heze, 274000 China; 3 Shenyang Medical College, No. 146 Road, Huanghe South Street, Huanggu District, Shenyang, 110034 China

**Keywords:** Lung cancer, Circulating tumor cells, *hTERT*, Radiotherapy, Efficacy

## Abstract

**Background:**

Circulating tumor cells (CTCs) are important biological indicators of the lung cancer prognosis, and CTC counting and typing may provide helpful biological information for the diagnosis and treatment of lung cancer.

**Methods:**

The CTC count in blood before and after radiotherapy was detected by the CanPatrol™ CTC analysis system, and the CTC subtypes and the expression of *hTERT* before and after radiotherapy were detected by multiple in situ hybridization. The CTC count was calculated as the number of cells per 5 mL of blood.

**Results:**

The CTC positivity rate in patients with tumors before radiotherapy was 98.44%. Epithelial–mesenchymal CTCs (EMCTCs) were more common in patients with lung adenocarcinoma and squamous carcinoma than in patients with small cell lung cancer (P = 0.027). The total CTCs (TCTCs), EMCTCs, and mesenchymal CTCs (MCTCs) counts were significantly higher in patients with TNM stage III and IV tumors (P < 0.001, P = 0.005, and P < 0.001, respectively). The TCTCs and MCTCs counts were significantly higher in patients with an ECOG score of > 1 (P = 0.022 and P = 0.024, respectively). The TCTCs and EMCTCs counts before and after radiotherapy affected the overall response rate (ORR) (P < 0.05). TCTCs and ECTCs with positive *hTERT* expression were associated with the ORR of radiotherapy (P = 0.002 and P = 0.038, respectively), as were TCTCs with high *hTERT* expression (P = 0.012). ECOG score (P = 0.006) and post-radiation TCTCs count (P = 0.011) were independent factors for progression-free survival (PFS) and TNM stage (P = 0.054) and pre-radiation EMCTCs count (P = 0.009) were independent factors of overall survival (OS).

**Conclusion:**

This study showed a high rate of positive CTC detection in patients with lung cancer, and the number, subtype, and *hTERT*-positive expression of CTCs were closely related to patients’ ORR, PFS, and OS with radiotherapy. EMCTCs, *hTERT*-positive expression of CTCs are expected to be important biological indicators for predicting radiotherapy efficacy and the prognosis in patients with lung cancer. These results may be useful in improving disease stratification for future clinical trials and may help in clinical decision-making.

**Supplementary Information:**

The online version contains supplementary material available at 10.1186/s12885-023-10979-z.

## Background

According to the latest data from the World Health Organization in 2020, lung cancer is still the leading cause of cancer-related death, with an estimated 1.8 million deaths (18.0% of all cancer-related deaths), followed by colorectal cancer (9.4%), hepatic and rectal cancer (8.3%), stomach cancer (7.7%), and female breast cancer (6.9%). Lung cancer is the leading malignant tumor in China in terms of incidence and mortality, and it is extremely harmful to human health [1, 2] Most patients are already in an advanced stage when they are diagnosed and therefore miss the chance for surgical treatment. With intensive basic and clinical research, the understanding of pathogenic genomic alterations in lung cancer has improved. This has in turn facilitated the development of new drugs and clinical use of biomarkers, resulting in significant progress in lung cancer treatment [3]. The combination of targeted therapy, immunotherapy, and advanced radiotherapy techniques has significantly improved survival and brought new hope to patients with lung cancer. Building on this foundation to improve follow-up monitoring of disease and comprehensive patient management may further improve treatment strategies and enhance treatment efficacy [4]. Pulmonary fibrosis after radiotherapy has been found to be difficult to distinguish from tumor recurrence on computed tomography during patient follow-up; in addition, micro-metastases of tumor cells are difficult to detect on computed tomography examination. Therefore, it is possible that the evaluation of radiotherapy efficacy using computed tomography examination results is not very accurate, which increases the difficulty of developing precise and individualized treatment strategies. If a reliable, noninvasive, and continuous low-risk biological index reflecting the systemic state of the disease can be found, it will inevitably provide important help for monitoring, diagnosing, and predicting treatment efficacy. This is a practical issue of high concern to clinicians.

As new biomarkers, circulating tumor cells (CTCs) are of great interest in medical research. “CTCs” is a collective term for the presence of traces of various types of tumor cells in the peripheral blood; these tumor cells originate from primary or metastatic foci and are the most direct factor in tumor recurrence and metastasis. The concept of CTCs was clarified by Paget in 1889 [5]. Subsequent studies showed that CTCs can be used to predict progression-free survival (PFS) and overall survival (OS) of patients with tumors, can be used to evaluate treatment efficacy [6] can contribute to the prediction and staging of tumor recurrence and metastasis, can help in drug screening and determination of therapeutic regimens for individualized tumor treatment [7], are complementary tests to traditional imaging and other biomarkers, can help to identify new tumor markers and develop new anti-tumor drugs, and are a new prognostic determinant for non-small cell lung cancer (NSCLC). In a study of CTC masses by Harvard University published in *Cell* [8], researchers found that CTC masses are formed by monoclonal tumor cell populations rather than aggregates in the circulation using a mouse model of tracer-labeled mammary tumors. Although CTC clusters in the blood are relatively rare compared with individual CTCs, they have 23 to 50 times the metastatic potential of individual CTCs. This study suggests that CTC clusters have high metastatic potential. Research has also shown that malignant cells lose some epithelial phenotypes (including morphology, surface antigens, and gene expression) and acquire some mesenchymal phenotypes in order to acquire motility and invasiveness. This is known as epithelial–mesenchymal transition (EMT), and most malignant cells undergo EMT during the process of detachment from the primary site. A recent study published in *Science* [9] showed that CTC masses are strongly associated with the mesenchymal phenotype and that a high proportion of CTCs with the mesenchymal phenotype exhibit chemoresistance. This shows that the clinical significance of different morphologies and classes of CTCs varies. However, most CTCs undergo apoptosis in the process of peripheral circulation or are directly phagocytosed by blood cells; only a few escape and develop metastatic foci. This suggests that the isolation, identification, and study of CTCs with the most metastatic potential are crucial.

The CanPatrol™ assay system (SurExam, Guangzhou, China) [10, 11] is a second-generation CTC enrichment technology based on immuno-removal combined with nano-filtration. It does not rely on specific antibody capture and has a removal efficiency of > 99.97% for leukocytes and a recovery rate of > 80% for tumor cells. The high recovery rate and the absence of reliance on surface antigens ensures the most comprehensive enrichment of CTCs in small-volume whole blood samples (5 mL) and, more importantly, CTCs with the highest metastatic potential (CTC masses and CTCs undergoing EMT). Enrichment of the most comprehensive range of CTCs is crucial for tumor recurrence surveillance and for noninvasive samples using CTCs as molecular assays. In addition, because CanPatrol™ CTCs are bound to filter membranes, they can be easily stored and flexibly analyzed for immunofluorescence, fluorescence in situ hybridization, molecular assays, and genome-wide analysis of individual CTCs by microdissection. CanPatrol™ CTC technology offers a new way of thinking and a new pathway for CTC enrichment. More comprehensive enrichment of CTCs provides technical assurance and better options for CTC research and clinical applications. The CanPatrol™ assay system has good sensitivity and specificity in detecting CTCs in peripheral blood of patients with NSCLC, which is valuable for assessment of the clinical prognosis [12].

Other studies have shown that telomerase activity is increased in almost all CTCs and that human telomerase reverse transcriptase (*hTERT*) is the determinant of telomerase activity [13]. Telomerase activity is correlated with *hTERT* overexpression during tumor development. *hTERT* expression is high in tumor cells but low or absent in normal tissues [14]. Patients with strong *hTERT* positivity have decreased 5-year survival rates, increased postoperative recurrence rates, a poorer prognosis, and shorter survival times. In clinical practice, further investigation is needed to identify the changes in the number and type of CTCs in patients with lung cancer before and after radiotherapy or radiochemotherapy, elucidate the relationship between radiotherapy efficacy and the type of CTCs, determine the expression of *hTERT* in CTCs, and clarify the relationship of CTCs with the sensitivity to radiotherapy.

The present study was performed to detect the changes in the CTC number, CTC type, and *hTERT* gene expression before and after lung cancer radiotherapy by multiplex RNA in situ hybridization; observe the local tumor changes by imaging; and analyze the relationship and significance of the CTC number, CTC subtype, and *hTERT* gene expression changes with radiotherapy efficacy by blood chemistry.

## Materials and methods

### Patients’ general information

We collected data from patients with lung cancer who visited the Radiotherapy Department of the General Hospital of Northern Theater Command for the first time for chest radiotherapy from January 2016 to May 2019. All patients had a clear pathological diagnosis and an indication for radiotherapy according to the treatment principles of the NCCN guideline. The study was approved by the Ethics Committee of the General Hospital of Northern Theater Command, and all patients provided written informed consent. All methods were performed in accordance with the relevant guidelines and regulations. Clinical efficacy was evaluated using the Response Evaluation Criteria In Solid Tumors (RECIST) 1.1 [15]. Five milliliters of anticoagulated peripheral blood was collected within 1 week before and 4 weeks after radiotherapy for CTC detection and *hTERT* analysis.

### Tumor cell enrichment

The CanPatrol™ CTC enrichment technique (SurExam, Guangzhou, China) was performed on peripheral blood samples to isolate and classify CTCs as previously described [10, 11]. A 5-mL peripheral blood sample was collected using EDTA-anticoagulated blood collection tubes and mixed upside down. Next, 15 mL of erythrocyte lysis solution was added, and the solution was mixed. The erythrocytes were lysed at room temperature for 30 min. The supernatant of the blood sample was then removed by centrifugation. The cell sediment was resuspended using phosphate-buffered saline (PBS) and fixed using 4% formaldehyde for 8 min. The fixed cells were then transferred to a filter tube (with filter membrane), and the cells were filtered onto the filter membrane using a vacuum suction pump. Fixation of the filtered cell membrane samples continued using 4% formaldehyde for 1 h at room temperature.

### Multiplex RNA in situ analysis

The fixed membrane samples were washed three times using PBS, placed in 24-well plates, treated with 0.1 mg/mL of proteinase K, and left at room temperature for 1 h to increase cell membrane permeability. They were then resuspended in PBS, and specific capture probes were added: epithelial biomarker probe (EpCAM, CK8/18/19), mesenchymal type biomarker probes (vimentin and Twist), leukocyte marker probes (CD45), and *hTERT* probes. The probes were hybridized in a hybridization reaction at 40 °C for 3 h. Unbound probes were washed three times with 1000 µL of eluent. Next, 100 µL of pre-amplification solution was added (formulation: 30% horse serum, 1.5% sodium dodecyl sulfate, 3 mM Tris-HCl (pH 8.0), 0.5 fmol pre-amplified probe), and incubation was performed at 40℃ for 30 min. The membrane was cooled, elution was performed with 1000 µL of eluent three times, 100 µL of amplification solution was added, 1 fmol of pre-amplified probe was added, and incubation was performed at 40 °C for 30 min. Four fluorescent proteins were added (see Table 1): the fluorescent dyes Alexa Fluor 594 (for the epithelial biomarker probes EpCAM and CK8/18/19), Alexa Fluor 488 (for the mesenchymal biomarker probes vimentin and Twist), and Alexa Fluor 750 (for labeling the leukocyte marker CD45), Cy7 (for biomarker probe *hTERT*). They were incubated at 40 °C for 30 min and eluted with 0.1×SSC, and 4’,6-diamidino-2-phenylindole (DAPI) was then used to stain the nuclei for 5 min. The samples were observed under 100× oil microscopy using an automated fluorescence scanning microscope. Red and green fluorescent signal dots represented epithelial and mesenchymal type gene expression on CTCs, respectively. The white signal points represented leukocyte marker CD45 gene expression. Purple fluorecent signal points represented *hTERT*gene expression.

**Table 1:** Capture probe sequences for the CD45, CK8, CK18, CK19, Ep-CAM, Vimentin, TWIST and hTERT gene

Gene	Sequences (5’-3’)
Ep-CAM	TGGTGCTCGTTGATGAGTCAAGCCAGCTTTGAGCAAATGAAAAGCCCATCATTGTTCTGGCTCTCATCGCAGTCAGGATCTCCTTGTCTGTTCTTCTGACCTCAGAGCAGGTTATTTCAG
CK8	CGTACCTTGTCTATGAAGGAACTTGGTCTCCAGCATCTTGCCTAAGGTTGTTGATGTAGCCTGAGGAAGTTGATCTCGTCCAGATGTGTCCGAGATCTGGTGACCTCAGCAATGATGCTG
CK18	AGAAAGGACAGGACTCAGGCGAGTGGTGAAGCTCATGCTGTCAGGTCCTCGATGATCTTGCAATCTGCAGAACGATGCGGAAGTCATCAGCAGCAAGACGCTGCAGTCGTGTGATATTGG
CK19	CTGTAGGAAGTCATGGCGAGAAGTCATCTGCAGCCAGACGCTGTTCCGTCTCAAACTTGGTTCTTCTTCAGGTAGGCCAGCTCAGCGTACTGATTTCCTCGTGAACCAGGCTTCAGCATC
Vimentin	GAGCGAGAGTGGCAGAGGACCTTTGTCGTTGGTTAGCTGGCATATTGCTGACGTACGTCAGAGCGCCCCTAAGTTTTTAAAAGATTGCAGGGTGTTTTCGGGCCAATAGTGTCTTGGTAG
TWIST	ACAATGACATCTAGGTCTCCTTTCAGTGGCTGATTGGCACTTACCATGGGTCCTCAATAACTGGTAGAGGAAGTCGATGCAACTGTTCAGACTTCTATCCCTCTTGAGAATGCATGCAT
CD45	TTACCATGGGTCCTCAATAATCGCAATTCTTATCGACTCTGTCATGGAGACAGTCATGTGTATTTCCAGCTTCAACTTCCCATCAATATAGCTGGCATTTTGTGCAGCAATGTATTTCC TACTTGAACCATCAGGCATC
*hTERT*	TATGTGGGGAGTGGAAGCCGAGTCAGCTTGAGCAGGAATGACATGCGTGAAACCTGTACGAAGGTGAGACTGGCTCTGATATACTCAGGGACACCTCGGAGCGTAGGAAGACGTCGAACACAGTACGTGTTCTGGGGTTTCTCTTCAAGTGCTGTCTGAT

*Ep-CAM* epithelial cell adhesion molecule, *CK* cytokeratins, *hTERT* human telomerase reverse transcriptase.

### Statistical analysis

Excel 2007 software was used to create the database. SPSS 20.0 software was used for statistical analysis. GraphPad Prism 7 was used for graphing of the data. Quantitative data with a skewed distribution are presented as median and 25th–75th quartiles. The Wilcoxon rank sum test was used for comparison of two groups, and the Kruskal–Wallis H test was used for comparison of multiple groups. Qualitative data are presented as relative numbers, and the χ^2^ test was used for comparison between groups. Differences were considered statistically significant at P < 0.05. The endpoint indicators in the clinical data analysis of this study were the overall response rate (ORR), OS, and PFS. ORR, OS, and PFS curves were plotted by the Kaplan–Meier method, and the effects of the variables on the prognosis were compared using the log-rank test.

## Results

### Characteristics of CTCs in patients with lung cancer

**Fig. 1:**
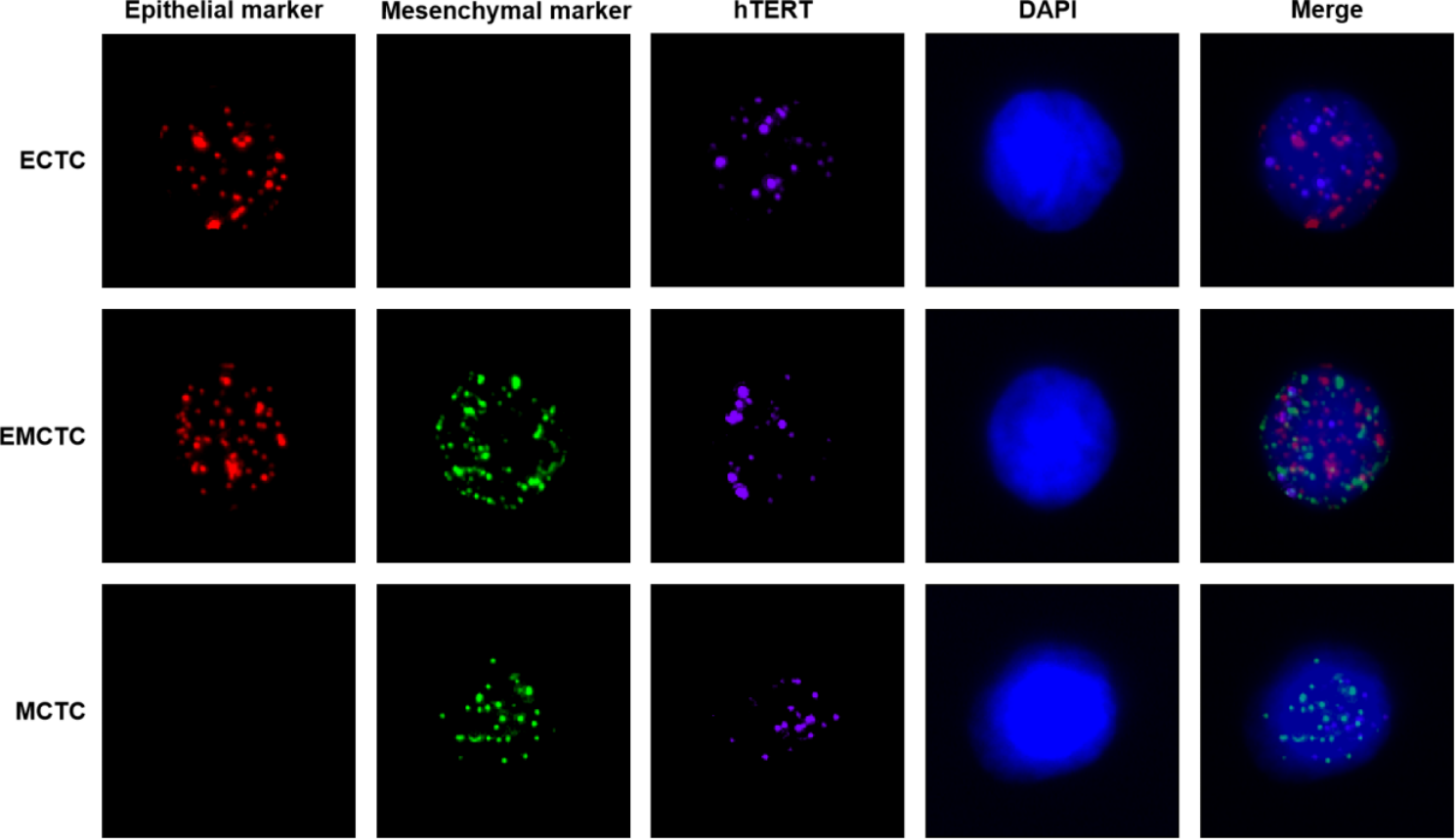
Expression of *hTERT* in CTC subtypes. The epithelial markers included EpCAM and CK 8/18/19, and the mesenchymal markers included vimentin and Twist **Abbreviations:** ECTC, epithelial circulating tumor cell; EMCTC, epithelial–mesenchymal circulating tumor cell; MCTC, mesenchymal circulating tumor cell.

In this experiment, 5 mL of venous blood was collected from the patients within 1 week before and 4 weeks after radiotherapy, and the number of CTCs in 5 mL of blood was detected by the CanPatrol™ CTC analysis system. Three subpopulations of CTCs were classified according to their immunofluorescence signals: epithelial CTCs (ECTCs), epithelial–mesenchymal CTCs (EMCTCs), and mesenchymal CTCs (MCTCs). ECTCs with only epithelial molecular markers (EpCAM and CK 8/18/19) were stained with red immunofluorescence, MCTCs with only mesenchymal molecular markers (vimentin and Twist) were stained with green immunofluorescence, and EMCTCs with two molecular markers were stained with green and red immunofluorescence (Fig. 1). The positive rate of CTCs in patients with tumors before radiotherapy was 98.44%. The total CTCs (TCTCs) count was 1113 (range, 0–104), and the positive rate of ECTCs was 68.75%, that of EMCTCs was 95.31%, and that of MCTCs was 76.56%. Details are shown in Supplementary Table 1.

### Relationship between CTC count and general clinical characteristics of 64 patients

64 patients with lung cancer were enrolled, including 32 with squamous cell carcinoma (SCC), 27 with adenocarcinoma (ADC) and 5 with small cell cancer (SCA). The median CTC count before radiotherapy was 13. The 64 patients were divided into 2 groups according to the median CTC count: those with a CTC count of ≤ 13 (34/64, 53.125%) and those with a CTC count of > 13 (30/64, 46.875%). The general clinical characteristics of the two groups were compared, and the results showed that the proportions of patients with TNM stage III and IV tumors, smoking, and tumors of > 4 cm were significantly higher among patients with a CTC count of > 13 than ≤ 13 (P < 0.001, P = 0.040, and P = 0.027, respectively). There were no statistically significant differences in the other clinicopathological characteristics between the two groups. Details are shown in Table 2.

**Table 2:** Relationship between CTC count and patients’ general characteristics

Factor	Total		CTCs number ≤ 13		CTCs number>13		*χ* ^2^	*P*
	N	Ratio (%)	N	Ratio (%)	N	Ratio (%)		
Age (years)							2.107	0.147
≤ 60	36	56.25	22	64.71	14	46.67		
>60	28	43.75	12	35.29	16	53.33		
Gender							0.323	0.570
Male	56	87.50	29	85.29	27	90.00		
Female	8	12.50	5	14.71	3	10.00		
Histology							2.016	0.365
SCC	32	50.00	15	44.12	17	56.67		
ADC	27	42.19	15	44.12	12	40.00		
SCA	5	7.81	4	11.76	1	3.33		
TNM Stage							14.337	<0.001^*^
I+II	19	29.69	17	50.00	2	6.67		
III + IV	45	70.31	17	50.00	28	93.33		
Chemotherapy							0.058	0.810
Yes	52	81.25	28	82.35	24	80.00		
No	12	18.75	6	17.65	6	20.00		
Surgical Operation							0.017	0.897
Yes	4	6.25	2	5.88	2	6.67		
No	60	93.75	32	94.12	28	93.33		
Smoking							4.205	0.040^*^
Yes	43	67.19	19	55.88	24	80.00		
No	21	32.81	15	44.12	6	20.00		
ECOG							2.824	0.093
≤ 1	41	64.06	25	73.53	16	53.33		
>1	23	35.94	9	26.47	14	46.67		
Tumor Size (cm)							4.916	0.027^*^
≤ 4	35	54.69	23	67.65	12	40.00		
>4	29	45.31	11	32.35	18	60.00		
CEA (ng/ml)							0.465	0.496
≤ 5	37	57.81	21	61.76	16	53.33		
>5	27	42.19	13	38.24	14	46.67		
NSE (ng/ml)							0.026	0.872
≤ 20	58	90.63	31	91.18	27	90.00		
> 20	6	9.38	3	8.82	3	10.00		
SCCA (ng/ml)							0.111	0.739
≤ 1.5	27	42.19	15	44.12	12	40.00		
> 1.5	37	57.81	19	55.88	18	60.00		

Notes:^*^P < 0.05.

**Abbreviations:** CTCs, circulating tumor cells; SCC, squamous cell carcinoma; ADC, adenocarcinoma; SCA, small cell cancer. (SCC); ECOG, Eastern Cooperative Oncology Group

### Relationship between CTC subgroups and general clinical characteristics of 64 patients

Analysis of the relationship between the presence of various phenotypic CTC subpopulations and clinicopathological features revealed that EMCTCs were more common in patients with lung adenocarcinoma and squamous carcinoma than in patients with small cell lung cancer (P = 0.027). The TCTC, EMCTC, and MCTC counts were significantly higher in patients with TNM stages III and IV tumors than in those with stages I and II (P < 0.001, P = 0.005, and P < 0.001, respectively). The TCTC and MCTC counts were significantly higher in patients with an ECOG score of > 1 than ≤ 1 (P = 0.022 and P = 0.024, respectively) (Table 3).

**Table 3:** Association of CTC subgroups with general clinical characteristics of 64 patients. Data are presented as median (interquartile range)

Groups	TCTCs (/5ml)	ECTCs	EMCTCs	MCTCs
SCC	14.00 (9.50,23.00)	1.00 (0.00,5.00)	6.00 (4.00,18.00)	3.00 (1.00,5.00)
ADC	13.00 (10.00,20.00)	3.00 (0.00,6.50)	6.00 (3.50,10.00)	2.00 (1.00,4.50)
SCA	9.00 (1.00,13.50)	2.00 (0.00,5.00)	2.00 (0.50,4.50)	1.00 (1.00,6.50)
*χ* ^2^	4.397	0.648	7.210	0.320
*P*	0.111	0.723	0.027*	0.852
TNM Stage				
I+II	8.00 (5.00,11.00)	2.00 (0.00,4.00)	4.00 (2.00,6.00)	1.00 (0.00,1.00)
III + IV	15.50 (11.00,24.25)	2.00 (0.00,5.75)	6.50 (4.00,17.00)	3.00 (2.00,5.75)
*Z*	-4.394	-0.508	-2.806	-4.071
*P*	0.000*	0.611	0.005*	<0.001*
ECOG				
≤ 1	11.00 (6.50,17.00)	3.00 (0.00,5.00)	6.00 (3.00,9.00)	1.00 (1.00,3.00)
>1	14.50 (12.00,27.50)	2.00 (0.00,6.25)	7.00 (3.00,19.75)	4.00 (2.00,7.25)
*Z*	-2.291	-0.377	-1.255	-2.265
*P*	0.022^*^	0.706	0.209	0.024^*^

**Notes**: ^*^P < 0.05: statistical description: median (figures outside the parentheses) and interquartile range (figures in the parentheses)

**Abbreviations:** CTCs, circulating tumor cells; ECTCs, epithelial circulating tumor cells; EMCTCs, epithelial–mesenchymal circulating tumor cells; MCTCs, mesenchymal circulating tumor cells; TCTCs, total CTCs. SCC, squamous cell carcinoma; ADC, adenocarcinoma; SCA, small cell cancer. (SCC)

### Changes in CTC count and *hTERT* expression before and after radiotherapy

The changes in the CTC count and *hTERT* expression in 64 patients before radiotherapy and 52 patients after radiotherapy were analyzed. *hTERT*gene expression was stained purple immunofluorescence (Fig. 1). The TCTC count before radiotherapy was 1113, and the *hTERT*-positive expression rate was 55.08%.The positive expression rate of *hTERT* was 43.42% in MCTCs, 63.39% in EMCTCs and 37.70% in ECTCs. The TCTC count after radiotherapy was 501, and the *hTERT*-positive expression rate was 58.88%. the MCTC count was 72 with an *hTERT*-positive expression rate of 44.44%, the ECTC count was 336 with an *hTERT*-positive expression rate of 65.77%, and the ECTC count was 93 with an *hTERT*-positive expression rate of 45.16%).

**Fig. 2:**
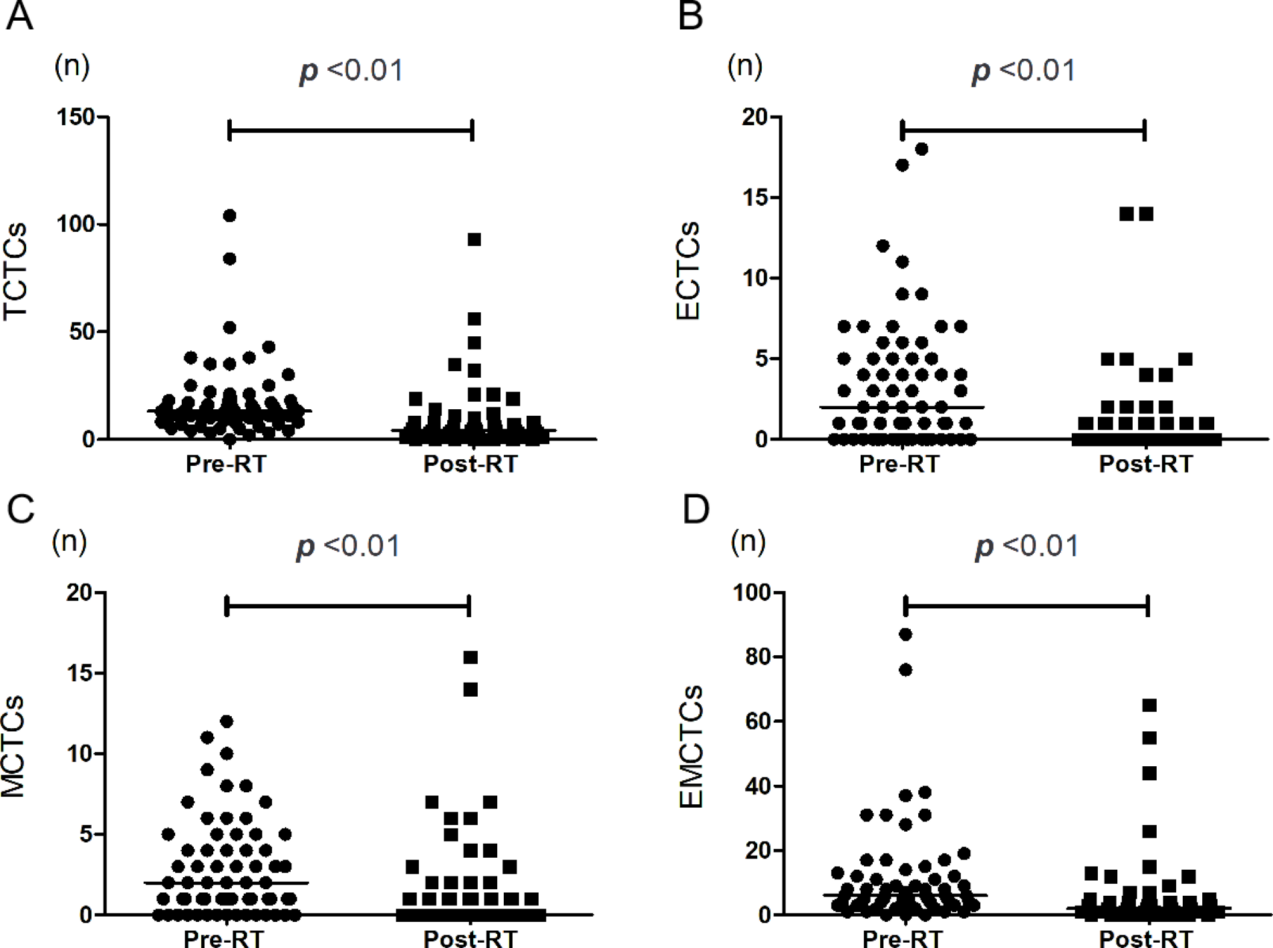
Changes in expression of CTCs before and after radiotherapy. (**a–d**) Changes in TCTC, ECTC, EMCTC, and MCTC counts before and after radiotherapy, P < 0.05 **Abbreviations:** Pre-RT, Pre-radiotherapy; Post-RT, Post-radiotherapy, ECTCs, epithelial circulating tumor cells; EMCTCs, epithelial–mesenchymal circulating tumor cells; MCTCs, mesenchymal circulating tumor cells; TCTCs, total CTCs.

**Fig. 3:**
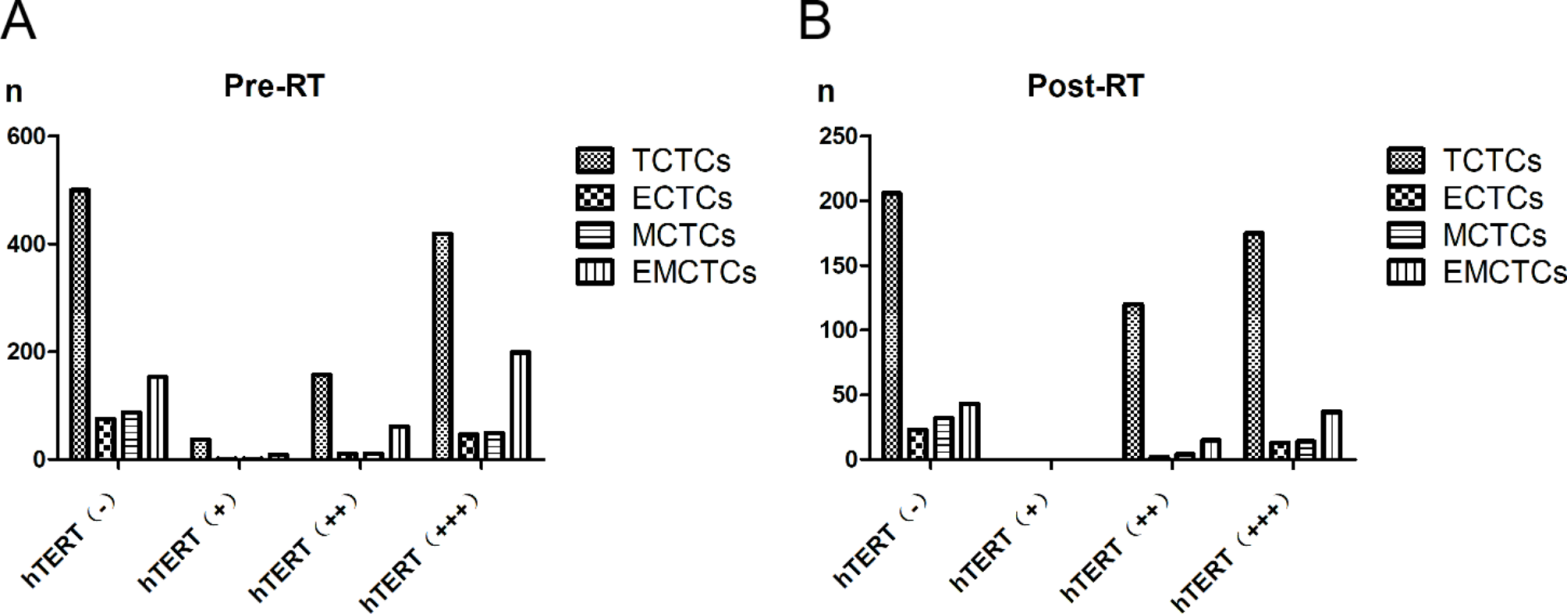
*hTERT* expression before and after radiotherapy. (**a**) *hTERT* expression before radiotherapy. (**b**) *hTERT* expression after radiotherapy **Abbreviations:** Pre-RT, Pre-radiotherapy; Post-RT, Post-radiotherapy, ECTCs, epithelial circulating tumor cells; EMCTCs, epithelial–mesenchymal circulating tumor cells; MCTCs, mesenchymal circulating tumor cells; TCTCs, total CTCs.

The TCTC, MCTC, and ECTC counts were significantly lower after than before radiotherapy (P < 0.01). The *hTERT*-positive expression rate also decreased, but the difference was not statistically significant (P > 0.05). The TCTC, MCTC, and ECTC counts with positive *hTERT* expression showed no statistically significant difference (P > 0.05). The changes in CTCs before and after radiotherapy are shown in Fig. 2, and the relationship of *hTERT* expression is shown in Fig. 3.

### Patients’ general clinical characteristics and relationship between number and subtypes of CTCs and efficiency of radiotherapy

The clinical efficacy among all 64 patients was evaluated before and after radiotherapy by reviewing computed tomography images and using RECIST 1.1. Three patients achieved a complete repose (CR), 35 achieved a partial response (PR), 25 had stable disease (SD), and 1 had progressive disease (PD). The ORR (CR + PR) was 59.375%. The general clinical characteristics and the relationship between the number and subtype of CTCs and radiotherapy efficiency were analyzed in all 64 patients, and the results showed that the TNM stage, ECOG score, and tumor size significantly affected the radiotherapy efficiency (P < 0.05) (Table 4) and that the TCTC and EMCTC counts before and after radiotherapy significantly affected ORR (P < 0.05) (Table 5). Further analysis of the relationship between the changes in the CTC count after radiotherapy and ORR revealed that the CTC count decreased in 45 patients after radiotherapy, and the treatment efficiency was 66.67%. Additionally, After radiotherapy, the number of CTCs increased in 11 patients, and the curative effect was evaluated. PR was found in 8 patients, and the therapeutic effective rate was 72.73%. Among the 8 patients, 4 patients developed radiation pneumonia, 3 patients developed tumor recurrence and metastasis during long-term follow-up, and 1 patient was stable.

**Table 4:** Relationship between patients’ general clinical characteristics and number and subtypes of CTCs and ORR

Characteristics	CR + PR (ORR)		SD + PD		*χ* ^2^	*P*
	N	Ratio (%)	N	Ratio (%)		
Age (years)					3.459	0.063
≤ 60	25	65.79	11	42.31		
>60	13	34.21	15	57.69		
Gender					1.814	0.178
Male	35	92.11	21	80.77		
Female	3	7.89	5	19.23		
Histology					3.939	0.139
SCC	17	44.74	15	57.69		
ADC	16	42.11	11	42.31		
SCA	5	13.16	0	0.00		
TNM Stage					6.910	0.009^*^
I+II	16	42.11	3	11.54		
III + IV	22	57.89	23	88.46		
Chemotherapy					1.920	1.166
Yes	33	86.84	19	73.08		
No	5	13.16	7	26.92		
Surgical Operation					0.155	0.693
Yes	2	5.26	2	7.69		
No	36	94.74	24	92.31		
Smoking					0.689	0.407
Yes	24	63.16	19	73.08		
No	14	36.84	7	26.92		
ECOG					6.100	0.014^*^
≤ 1	29	76.32	12	46.15		
>1	9	23.68	14	53.85		
Tumor Size (cm)					4.652	0.031^*^
≤ 4	25	65.79	10	38.46		
>4	13	34.21	16	61.54		
CEA (ng/ml)					0.282	0.595
≤ 5	23	60.53	14	53.85		
>5	15	39.47	12	46.15		
NSE (ng/ml)					0.146	0.702
≤ 20	34	89.47	24	92.31		
> 20	4	10.53	2	7.69		
SCCA (ng/ml)					4.183	0. 041^*^
≤ 1.5	20	52.63	7	26.92		
> 1.5	18	47.37	19	73.08		

Notes:^*^P < 0.05.

**Abbreviations:** CR, complete repose; PR, partial response. SD, stable disease, PD, progressive disease; SCC, squamous cell carcinoma; ADC, adenocarcinoma; SCA, small cell cancer. (SCC); ECOG, Eastern Cooperative Oncology Group; ORR, overall response rate, ORR = CR + PR

**Table 5:** Relationship between ORR and CTC subtypes

Factor	Treatment Effectiveness Evaluation		*Z*	*P*
	CR + PR (ORR)	SD + PD		
TCTCs				
Pre-RT	11.00 (6.50,14.50)	18.00 (14.00,27.50)	-4.176	<0.001^*^
Post-RT	3.00 (1.00,5.50)	7.50 (3.75,19.50)	-2.185	0.029^*^
ECTCs				
Pre-RT	3.00 (0.00,4.50)	1.50 (0.00,6.25)	-0.785	0.432
Post-RT	0.00 (0.00,1.00)	0.50 (0.00,2.50)	-0.698	0.485
EMCTCs				
Pre-RT	5.00 (2.50,7.00)	10.50 (4.75,20.50)	-3.268	0.001^*^
Post-RT	1.00 (0.00,4.00)	4.00 (2.00,9.75)	-2.418	0.016^*^
MCTCs				
Pre-RT	2.00 (1.00,4.00)	3.50 (1.75,6.25)	-1.644	0.100
Post-RT	0.00 (0.00,2.00)	1.00 (0.00,4.50)	-1.286	0.198

**Notes**: ^*^P < 0.05;statistical description: median (figures outside the parentheses) and interquartile range (figures in the parentheses)

**Abbreviations:** CR, complete repose; PR, partial response. SD, stable disease, PD, progressive disease; SCC, squamous cell carcinoma; Pre-RT, Pre-radiotherapy; Post-RT, Post-radiotherapy; ECTCs, epithelial circulating tumor cells; EMCTCs, epithelial–mesenchymal circulating tumor cells; MCTCs, mesenchymal circulating tumor cells; TCTCs, total CTCs; ORR, overall response rate, ORR = CR + PR.

### Relationship between *hTERT*-positive expression/high expression before radiotherapy and efficiency of radiotherapy

The relationship between *hTERT*-positive expression and radiotherapy efficiency before radiotherapy was analyzed in all 64 patients. The results showed that *hTERT*-positive expression of TCTCs and ECTCs was significantly associated with treatment efficiency (P = 0.002 and 0.038, respectively) and that *hTERT*-positive expression of EMCTCs had some effect on treatment efficacy, but the difference was not statistically significant (P = 0.052) (Table 6).

**Table 6:** Relationship between *hTERT*-positive expression expression before radiotherapy and ORR

Factor	CR + PR (ORR)		SD + PD		*χ* ^2^	*P*
	N	Ratio (%)	N	Ratio (%)		
*hTERT* (+) TCTCs					9.328	0.002^*^
≤ 5	25	65.79	7	26.92		
>5	13	34.21	19	73.08		
*hTERT* (+) MCTCs					0.855	0.355
= 0	22	57.89	12	46.15		
> 0	16	42.11	14	53.85		
*hTERT* (+) EMCTCs					3.781	0.052
≤ 3	24	63.16	10	38.46		
> 3	14	36.84	16	61.54		
*hTERT* (+) ECTCs					4.316	0.038^*^
= 0	26	70.27	11	42.31		
> 0	11	29.73	15	57.69		
*hTERT* Highly expressed TCTCs					6.385	0.012^*^
≤ 5	28	73.68	11	42.31		
> 5	10	26.32	15	57.69		
*hTERT* Highly expressed MCTCs					1.287	0.257
= 0	23	60.53	12	46.15		
> 0	15	39.47	14	53.85		
*hTERT* Highly expressed EMCTCs					3.781	0.052
≤ 2	24	63.16	10	38.46		
> 2	14	36.84	16	61.54		
*hTERT* Highly expressed ECTCs					3.42	0.064
= 0	25	67.57	12	44.44		
> 0	12	32.43	15	55.56		

Notes:^*^P < 0.05.

**Abbreviations:** CR, complete repose; PR, partial response. SD, stable disease, PD, progressive disease; SCC, squamous cell carcinoma; ECTCs, epithelial circulating tumor cells; EMCTCs, epithelial–mesenchymal circulating tumor cells; MCTCs, mesenchymal circulating tumor cells; TCTCs, total CTCs; ORR, overall response rate, ORR = CR + PR

The relationship between high *hTERT* expression before radiotherapy and radiotherapy efficiency was also analyzed in all 64 patients. The results showed that TCTCs with high *hTERT* expression were significantly associated with ORR (P = 0.012) and that EMCTCs and ECTCs with high *hTERT* expression also had an effect on ORR, but the difference was not statistically significant (Table 6).

### Prognostic analysis of patients’ general clinical characteristics and CTC-related characteristics

Among all 64 patients, the mean follow-up time after radiotherapy was 20.73 (4.7–47.6) months, the median PFS was 14.830 (1.33–37.77) months, and the median OS was 40.230 (4.7–47.5) months. Univariate prognostic analysis of all patients suggested that the relevant factors affecting PFS were pathology (P = 0.002), TNM stage (P < 0.001), ECOG score (P < 0.001), tumor size (P = 0.002), pre-radiotherapy TCTC count (P = 0.003), post-radiotherapy TCTC count (P = 0.001), pre-radiotherapy EMCTC count (P = 0.035), post-radiotherapy EMCTC count (P = 0.030), and post-radiotherapy ECTC count (P = 0.033). Univariate prognostic analysis also suggested that the relevant factors affecting OS were pathology (P = 0.002), TNM stage (P = 0.017), ECOG score (P = 0.040), pre-radiotherapy TCTC count (P = 0.023), pre-radiotherapy EMCTC count (P = 0.002), and pre-radiotherapy *hTERT*-positive EMCTC count (P = 0.046) (see Table 7 for details). The factors with a P value of < 0.05 in the univariate analysis were included in the Cox model for multifactor analysis, and the results showed that independent factors influencing PFS for patients with lung cancer were the ECOG score (P = 0.006) and post-radiation TCTC count (P = 0.011) and that those influencing OS were the TNM stage (P = 0.054) and pre-radiation EMCTC count (P = 0.009) (Tables 8 and 9).

**Table 7:** Log-rank univariate OS and PFS analysis of 64 patients with lung cancer

Factor	mOS (95%CI)	*P*	mPFS (95%CI)	*P*
Age (years)		0.365		0.289
≤ 60	40.230 (34.731–45.729)		14.900 (3.498–26.302)	
>60	—		14.340 (10.084–18.596)	
Gender		0.999		0.339
female	42.930 (—)		10.630 (1.580–19.680)	
male	40.230 (34.778–45.682)		14.900 (5.947–23.853)	
Histology		0.219		0.002^*^
SCC	37.930 (37.249–38.611)		14.500 (1.271–27.729)	
ADC	42.930 (21.276–64.584)		21.470 (8.098–34.842)	
SCA	26.000 (—)		5.500 (0.000-11.233)	
TNM Stage		0.017*		0.001^*^
I/II	43.470 (—)		28.270 (25.892–30.648)	
III/IV	37.930 (18.560–57.300)		12.870 (8.561–17.179)	
Smoking		0.876		0.584
Yes	42.930 (—)		14.830 (0.323–29.337)	
No	40.230 (34.663–45.797)		14.900 (9.193–20.607)	
ECOG		0.040*		0.000^*^
≤ 1	—		27.500 (22.864–32.136)	
> 1	37.930 (10.296–65.564)		11.233 (7.834–14.632)	
Tumor Size (cm)		0.092		0.002^*^
≤ 4	42.930 (38.301–47.559)		24.000 (8.021–39.979)	
>4	37.630 (4.625–70.635)		10.630 (7.219–14.041)	
TCTCs of Pre-RT (n)		0.023*		0.003^*^
≤ 13	40.230 (20.293–60.167)		27.470 (20.882–34.058)	
>13	37.630 (13.424–61.836)		11.700 (7.229–16.171)	
TCTCs of Post-RT (n)		0.173		0.001^*^
≤ 4	42.930 (37.675–48.185)		27.500 (18.045–36.955)	
>4	37.630 (1.823–73.437)		10.100 (6.287–13.913)	
ECTCs of Pre-RT (n)		0.144		0.233
≤ 2	43.470 (—)		24.000 (10.728–37.272)	
>2	37.930 (23.548–52.312)		14.000 (10.912–17.088)	
ECECs of Post-RT (n)		0.099		0.033^*^
= 0	42.930 (33.760–52.100)		27.470 (14.189–40.751)	
>0	40.230 (16.614–63.846)		10.467 (6.818–14.115)	
EMCTCs of Pre-RT (n)		0.002*		0.035^*^
≤ 6	47.400 (35.847–58.953)		24.000 (7.346–40.654)	
>6	37.630 (9.675–65.585)		12.500 (3.665–21.335)	
EMCTCs of Post-RT (n)		0.516		0.030^*^
≤ 2	40.230 (25.837–54.623)		26.500 (12.373–40.627)	
>2	37.930 (32.652–43.208)		10.630 (6.384–14.876)	
*hTERT* (+) TCTCs (n) of Pre-RT		0.106		0.520
≤ 5	40.230 (14.691–65.769)		22.500 (8.698–36.302)	
>5	37.930 (32.248–43.612)		14.000 (9.452–18.548)	
*hTERT* (+) EMCTCs (n) of Pre-RT		0.046*		0.206
≤ 3	40.230 (20.207–60.253)		24.000 (8.407–39.593)	
>3	37.930 (12.235–63.625)		14.340 (9.603–19.077)	
*hTERT* (+) ECTCs (n) of Pre-RT		0.515		0.462
= 0	43.470 (—)		23.46 (9.444–37.490)	
>0	40.230 (36.355–44.105)		14.340 (13.036–15.644)	

Notes:^*^P < 0.05.

**Abbreviations:** CI, confidence index; OS, overall survival; PFS, progression-free survival; SCC, squamous cell carcinoma; ECTCs, epithelial circulating tumor cells; EMCTCs, epithelial–mesenchymal circulating tumor cells; MCTCs, mesenchymal circulating tumor cells; TCTCs, total CTCs; Pre-RT, Pre-radiotherapy; Post-RT, Post-radiotherapy

**Table 8:** Multifactor PFS analysis of Cox model in 64 patients with lung cancer

Foctor	PFS		
	HR (95%CI)		*P*
ECOG	3.592 (1.495–8.630)		0.006^*^
TCTCs of Post-RT	2.783 (1.316–5.888)		0.011^*^

Notes:^*^P < 0.05.

**Abbreviations:** CI, confidence index; PFS, progression-free survival; TCTCs, total CTCs; Post-RT, Post-radiotherapy.

**Table 9:** Multifactor OS analysis of Cox model in 64 patients with lung cancer

Factor	OS	
	HR (95%CI)	*P*
TNM Stage	7.677 (0.968–60.890)	0.054^*^
EMCTCs (n) of Pre-RT	4.910 (1.484–16.244)	0.009^*^

Notes:^*^P < 0.05.

**Abbreviations:** CI, confidence index; OS, overall survival; EMCTCs, epithelial–mesenchymal circulating tumor cells; Pre-RT, Pre-radiotherapy

## Discussion

Cancer is the first or second most common contributor to premature death in most countries of the world. The number of patients with cancer worldwide is expected to continue increasing during the next 50 years as demographic changes such as population aging and growth strongly influence different trends in cancer incidences in different regions. Lung cancer is one of the most common cancers and the leading cause of cancer-related deaths worldwide, with approximately 18% of cancer-related deaths associated with lung cancer [1]. Thus, lung cancer poses a serious threat to human health. Lung cancer is histologically divided into SCLC and NSCLC, with NSCLC accounting for approximately 85% of lung malignancies [16]. The early diagnosis rate of lung cancer is about 15%, and 75% of patients are diagnosed with locally advanced or late-stage cancer [17]. With the increasing understanding of the biological features of this disease, the use of predictive biomarkers, and improvements in treatment, significant progress has been made and survival has been prolonged [18]. However, patients with advanced lung cancer have a poor prognosis, and despite the continued clinical citation of new specific checkpoint inhibitors, the 5-year survival rate remains below 15% [19]. Therefore, the identification of an easily accessible, specific, and sensitive tool for lung cancer diagnosis and prognosis will provide a new direction for the diagnosis and treatment of lung cancer.

CTCs are tumor cells in a free state that are shed from the primary or metastatic foci of a tumor and enter the peripheral circulation. They play an important role in tumorigenesis and progression. CTCs in patients with advanced NSCLC are associated with lower treatment response rates and shorter PFS and OS [20]. CTCs have been studied in depth, and it was found that single CTCs or heterotypic clusters are more significant predictors of the risk of disease recurrence in patient cohorts with early (stage I–II) and advanced (stage III–IVA) cancer, respectively [21]. CTCs are more likely to be detected in patients with advanced tumors, patients with positive CTCs in peripheral blood at baseline and after treatment have a poorer prognosis, and the rate of positive CTCs is correlated with the pretreatment tumor stage [22]. Analysis of data from patients with NSCLC revealed that CTCs are associated with tumor size, lymph node involvement, and distant metastasis but are not significantly correlated with histopathology, sex, or age [23]. Analysis of patients with SCLC revealed that CTC counts were closely related to clinical factors such as TNM stage, age, and the serum tumor marker neuron-specific enolase; higher CTCs counts were associated with a worse prognosis; and CTCs had a better predictive effect on the prognosis of SCLC [24]. Tay et al. [25] found that CTC count thresholds of 2, 15, and 50 at baseline for limited-stage SCLC were significantly associated with PFS and OS, with 15 being associated with worse PFS and OS and predicting ≤ 2 years of survival. We found that the positive rate of CTCs in patients with tumors before radiotherapy was 98.44%, and we defined a median CTC count of 13. We then grouped the patients according to the median CTC count of 13 and found that there were significantly more patients with stage III and IV tumors, smoking, and tumors of > 4 cm among patients with a CTC count of > 13 than ≤ 13 (P < 0.05). The other clinicopathological characteristics were not significantly related to the status of CTCs, suggesting that locally advanced or late-stage tumors, smoking, and tumor size are related to the CTC count and that patients with a CTC count of > 13 may be more likely to be diagnosed with recurrence and metastasis.

However, the use of CTC counts alone as an indicator for clinically meaningful assessment is still far from adequate. In fact, CTC counts alone cannot provide sufficient information to assess the status of patients with cancer [26]. Therefore, we further explored the relationship between CTC subpopulations and clinicopathological features. We detected CTCs in 5 mL of blood using the CanPatrol™ analysis system and distinguished CTC subgroups according to the in situ immunohybridization technique. ECTCs, EMCTCs, and MCTCs had positive rates of 68.75%, 95.31%, and 76.56%, respectively, and greater EMCTC positivity was seen in lung cancer, especially in adenocarcinoma and squamous carcinoma. Notably, the small number of patients with SCLC enrolled in this study may have introduced bias, and more patients with SCLC need to be enrolled for analysis in future studies. The TCTC, EMCTC, and MCTC counts were significantly higher in patients with TNM stage III and IV tumors in this study than in patients with stage I and II, similar to the results of most previous studies. One study showed that a CTC count of ≥ 16 and MCTC percentage of ≥ 2% preoperatively were significantly associated with early recurrence and metastasis [27]. EMT inhibits CTC apoptosis and makes CTCs difficult to recognize in the hematopoietic microenvironment, resulting in immune escape [28, 29]. The mesenchymal subtype promotes the movement of CTCs but is detrimental to proliferation. Tumor epithelial cells undergo EMT and acquire invasive metastatic capacity. Therefore, EMCTCs are more aggressive than ECTCs and more proliferative than MCTCs [30]. However, tumor cell migration does not always require phenotypic transformation, and the formation of CTC clusters also contributes significantly to the metastatic spread of cancer, but is relatively rare [8]. No CTC clusters were found in our study. In most patients with NSCLC, the EMCTC count is higher than the ECTC and MCTC counts, and organ metastasis is positively correlated with the TCTC, EMCTC, and MCTC counts [30]. The development of tumor invasiveness and metastasis can be accelerated when the dominant CTC subpopulation are E-CTCs with a hybrid E/M phenotype [31]. Studies of patients with liver cancer, however, have shown that CTC counts and the EMT phenotype are not associated with patient characteristics (e.g., age, sex, ECOG score, tumor number and size, vascular infiltration, tumor size) [32]. However, we found that the TCTC andMCTC counts were significantly higher in patients with ECOG scores of > 1 than ≤ 1. The results suggest that patients with higher ECOG score may be more likely to develop tumor metastasis. An ECOG score of 2 or 3 is an independent risk factor for a poor prognosis in patients with tumors [33] and the CTC and MCTC counts were also associated with prognostic factors in patients with tumors in our study. Therefore, it is reasonable that patients with high TCTC and MCTC counts had correspondingly high ECOG scores in our study.

TCTC, MCTC, MECTC, and ECTC counts were significantly lower after radiotherapy than before radiotherapy. Preliminarily, it was shown that radiotherapy affected the decrease in the CTC count in patients’ blood, and whether the rate of this decrease in the CTC count could predict the treatment efficacy requires further analysis. One study showed that CTC counts and EMT phenotypes in patients before treatment do not predict short-term efficacy and may correlate with long-term efficacy [32]. We also further analyzed the relationship between the changes in CTC counts and subtypes before and after radiotherapy and the efficacy of radiotherapy. The results suggested that the TNM stage, ECOG score, tumor size, TCTC count before and after radiotherapy, and EMCTC count all had significant effects on the ORR, suggesting that CTC counts and subtypes may be effective predictors of near-term efficacy. ORR is a necessary prerequisite for improving local control rate and prolonging survival in cancer patients. However, we performed a detailed analysis of different subtypes and found a relationship between EMCTCs and ORR, contradicting the previously reported lack of a correlation between CTCs and the treatment response. This discrepancy may be related to the different treatment modalities, and the sample size must be increased to further confirm the results of this study. In addition, we were surprised to find that 11 patients to show an increased number of CTC after radiotherapy, with a higher treatment efficiency (72.73%) compared to the patients with a decrease in the number of CTCs. There were 4 out of 11 patients developed III/IV radiation pneumonia. Therefore, the analysis considered that radiation pneumonia could affect the number of CTCs after radiotherapy, and the recent efficacy evaluation of these 4 patients was PR and PFS of 2.53–36.4 months. The current results cannot explain the relationship between the occurrence of radiation pneumonia and the prognosis of patients.

Several reports have focused on the correlation between the CTC count and the PFS and OS. Tay et al. [25]reported independent influences on PFS and OS in patients with SCLC who had CTC counts of ≥ 15, and they showed that CTC counts of ≥ 15 at baseline independently predicted ≤ 1 year of survival in 70% of patients and ≤ 2 years of survival in 100% of patients. Punnoose et al. [34] found that higher baseline CTC counts and decreased CTC counts after treatment were associated with a good treatment response and longer PFS. CTC levels are also considered to be prognostic markers for nasopharyngeal, prostate, breast, and colorectal cancers [35–39]. However, few studies have explored the correlation between CTC subgroups and the PFS and OS. One small-sample study in a Chinese population showed that OS was significantly correlated with the TCTC, ECTC, and EMCTC counts [40]. In our study, 64 patients were enrolled for post-radiotherapy follow-up with a mean follow-up time of 20.73 months, median PFS of 14.83 months, and median OS of 40.23 months. A univariate analysis of the variables affecting PFS and OS was performed, showing that the relevant factors affecting PFS were pathology, TNM stage, ECOG score, tumor size, pre-radiotherapy TCTC count, post-radiotherapy TCTC count, pre-radiotherapy EMCTC count, post-radiotherapy EMCTC count, and post-radiotherapy ECTC count. Additionally, the relevant factors affecting OS were the pathology, TNM stage, ECOG score, pre-radiotherapy TCTC count, and pre-radiotherapy EMCTC count. Among these, the ECOG score and post-radiotherapy TCTC count were independent influencing factors of PFS, and the TNM stage and pre-radiotherapy EMCTC count were independent influencing factors of OS in patients with lung cancer. This provides a new direction for the identification of biological treatment indicators for survival prediction in patients with NSCLC. However, studies with larger sample sizes are still needed to further explore the prognostic value of CTC subgroups.

Telomerase is silent in most differentiated human cells, mainly because of transcriptional repression of its catalytic component, the telomerase reverse transcriptase (TERT) gene. Studies have shown that TERT/telomerase activation is essential for cell immortalization and malignant transformation by stabilizing the telomere length and eliminating barriers to senescence [41]. TERT expression/telomerase activity can be detected in up to 90% of primary human cancers. Several basic studies have shown that telomerase is the first reactive protein during rapid tumor development, growth, and invasion [42]. Zhang N et al. [43] demonstrated that inhibition of telomerase expression will effectively inhibit human tumor cell growth. Ferrandon S et al. [44]suggested that high telomerase activity is a unique feature of tumor stem cells and that inhibition of telomerase expression may improve the efficacy of radiotherapy. In this study, we found that *hTERT*-positive expression of TCTCs and ECTCs was closely correlated with treatment efficiency, and the difference was statistically significant. *hTERT*-positive expression of EMCTCs affected efficiency but did not reach statistical significance, and patients with high *hTERT* expression of MECTCs with a count of > 5 had significantly poorer treatment efficiency. The total number of *hTERT*-expressing EMCTCs before radiotherapy was a relevant factor affecting OS. Therefore, *hTERT* may be an important biological marker to predict the sensitivity of radiotherapy.

## Conclusion

In summary, this study revealed a high rate of positive CTC detection in patients with lung cancer, and the number, subtype, and *hTERT*-positive expression of CTCs were closely related to patients’ ORR, PFS, and OS with radiotherapy. EMCTs, *hTERT*-positive expression, and high expression of CTCs are expected to be important biological indicators for predicting radiotherapy efficacy and the prognosis in lung cancer. These results may improve disease stratification in future clinical trials and help in clinical decision-making.

## Electronic supplementary material

Below is the link to the electronic supplementary material.

Supplementary Material 1

## Data Availability

The datasets used and analyzed in this study are available from the corresponding author upon reasonable request.
