# Supplementary material for: Circulating tumor cells (CTCs) and hTERT gene expression in CTCs for radiotherapy effect with lung cancer
Source: BMC Cancer. 2023 May 24;23:475. doi: 10.1186/s12885-023-10979-z (PMC10207832; doi:10.1186/s12885-023-10979-z)
Supplement: Supplementary file 1 — Supplementary Material 1 [file 12885_2023_10979_MOESM1_ESM.docx]

**Supplementary Table 1.** Relationship between CTC count before and after radiotherapy

| No. | Pre-RT(n) | | | Post-RT(n) | | |
| --- | --- | --- | --- | --- | --- | --- |
|  | ECTCs | EMCTCs | MCTCs | ECTCs | EMCTCs | MCTCs |
| 1 | 5.0 | 3.0 | 3.0 | 1.0 | 1.0 | 2.0 |
| 2 | 9.0 | 8.0 | 5.0 | 14.0 | 5.0 | 0.0 |
| 3 | 0.0 | 5.0 | 3.0 | 0.0 | 1.0 | 0.0 |
| 4 | 2.0 | 31.0 | 5.0 | 0.0 | 4.0 | 6.0 |
| 5 | 12.0 | 1.0 | 0.0 | - | - | - |
| 6 | 5.0 | 3.0 | 3.0 | 0.0 | 0.0 | 0.0 |
| 7 | 0.0 | 6.0 | 5.0 | 0.0 | 5.0 | 0.0 |
| 8 | 0.0 | 5.0 | 0.0 | 1.0 | 12.0 | 6.0 |
| 9 | 0.0 | 15.0 | 0.0 | - | - | #NULL! |
| 10 | 0.0 | 12.0 | 3.0 | 5.0 | 12.0 | 4.0 |
| 11 | 0.0 | 9.0 | 5.0 | 0.0 | 1.0 | 0.0 |
| 12 | 3.0 | 9.0 | 3.0 | 0.0 | 1.0 | 2.0 |
| 13 | 7.0 | 3.0 | 2.0 | 0.0 | 1.0 | 0.0 |
| 14 | 3.0 | 6.0 | 6.0 | 1.0 | 1.0 | 0.0 |
| 15 | 1.0 | 4.0 | 8.0 | 1.0 | 2.0 | 1.0 |
| 16 | 18.0 | 6.0 | 6.0 | 0.0 | 3.0 | 0.0 |
| 17 | 0.0 | 31.0 | 4.0 | 2.0 | 1.0 | 3.0 |
| 18 | 0.0 | 0.0 | 0.0 | 4.0 | 4.0 | 0.0 |
| 19 | 4.0 | 6.0 | 1.0 | 2.0 | 26.0 | 4.0 |
| 20 | 3.0 | 1.0 | 2.0 | 0.0 | 0.0 | 0.0 |
| 21 | 0.0 | 28.0 | 10.0 | 0.0 | 0.0 | 0.0 |
| 22 | 4.0 | 2.0 | 1.0 | 0.0 | 2.0 | 0.0 |
| 23 | 1.0 | 8.0 | 2.0 | 2.0 | 5.0 | 5.0 |
| 24 | 9.0 | 14.0 | 2.0 | 0.0 | 44.0 | 1.0 |
| 25 | 4.0 | 6.0 | 1.0 | 0.0 | 0.0 | 0.0 |
| 26 | 5.0 | 19.0 | 1.0 | 1.0 | 0.0 | 0.0 |
| 27 | 0.0 | 37.0 | 6.0 | 4.0 | 15.0 | 16.0 |
| 28 | 6.0 | 4.0 | 1.0 | 1.0 | 3.0 | 0.0 |
| 29 | 2.0 | 1.0 | 0.0 | 0.0 | 0.0 | 0.0 |
| 30 | 0.0 | 1.0 | 1.0 | 0.0 | 55.0 | 1.0 |
| 31 | 1.0 | 8.0 | 1.0 | - | - | - |
| 32 | 1.0 | 17.0 | 3.0 | 0.0 | 7.0 | 1.0 |
| 33 | 5.0 | 9.0 | 2.0 | 1.0 | 13.0 | 7.0 |
| 34 | 7.0 | 2.0 | 0.0 | 14.0 | 65.0 | 14.0 |
| 35 | 2.0 | 3.0 | 7.0 | 1.0 | 0.0 | 0.0 |
| 36 | 0.0 | 11.0 | 0.0 | 1.0 | 1.0 | 1.0 |
| 37 | 1.0 | 17.0 | 0.0 | 0.0 | 5.0 | 2.0 |
| 38 | 3.0 | 4.0 | 1.0 | 0.0 | 1.0 | 0.0 |
| 39 | 11.0 | 7.0 | 3.0 | 5.0 | 2.0 | 7.0 |
| 40 | 0.0 | 3.0 | 0.0 | 0.0 | 4.0 | 1.0 |
| 41 | 4.0 | 0.0 | 1.0 | 0.0 | 0.0 | 0.0 |
| 42 | 0.0 | 4.0 | 0.0 | 5.0 | 1.0 | - |
| 43 | 1.0 | 13.0 | 0.0 | 1.0 | 9.0 | 1.0 |
| 44 | 1.0 | 2.0 | 1.0 | 0.0 | 0.0 | 0.0 |
| 45 | 0.0 | 31.0 | 4.0 | 0.0 | 4.0 | 0.0 |
| 46 | 0.0 | 5.0 | 0.0 | 1.0 | 3.0 | 0.0 |
| 47 | 4.0 | 76.0 | 4.0 | - | - | - |
| 48 | 7.0 | 2.0 | 1.0 | 1.0 | 1.0 | 1.0 |
| 49 | 0.0 | 5.0 | 12.0 | 0.0 | 1.0 | 0.0 |
| 50 | 4.0 | 5.0 | 1.0 | 0.0 | 1.0 | 3.0 |
| 51 | 7.0 | 6.0 | 4.0 | 0.0 | 2.0 | 2.0 |
| 52 | 3.0 | 8.0 | 5.0 | - | - | - |
| 53 | 2.0 | 4.0 | 0.0 | - | - | - |
| 54 | 1.0 | 3.0 | 11.0 | - | - | - |
| 55 | 2.0 | 6.0 | 0.0 | - | - | - |
| 56 | 6.0 | 4.0 | 4.0 | 0.0 | 0.0 | 0.0 |
| 57 | 0.0 | 5.0 | 8.0 | 1.0 | 0.0 | 0.0 |
| 58 | 7.0 | 38.0 | 7.0 | 0.0 | 7.0 | 1.0 |
| 59 | 17.0 | 87.0 | 0.0 | - | - | - |
| 60 | 6.0 | 0.0 | 1.0 | - | - | - |
| 61 | 1.0 | 8.0 | 9.0 | 2.0 | 2.0 | 1.0 |
| 62 | 5.0 | 12.0 | 1.0 | - | - | - |
| 63 | 1.0 | 11.0 | 1.0 | - | - | - |
| 64 | 0.0 | 17.0 | 2.0 | 0.0 | 3.0 | 0.0 |

**Abbreviations:** Pre-RT, Pre-radiotherapy; Post-RT, Post-radiotherapy, ECTCs, epithelial circulating tumor cells; EMCTCs, epithelial–mesenchymal circulating tumor cells; MCTCs, mesenchymal circulating tumor cells.
